# Dietary Behavior and Compliance to Bulgarian National Nutrition Guidelines in Patients With Type 1 Diabetes With Longstanding Disease

**DOI:** 10.3389/fnut.2022.900422

**Published:** 2022-07-08

**Authors:** Rouzha Pancheva, Lyubomir Dimitrov, Michal Gillon-Keren, Kaloyan Tsochev, Tatyana Chalakova, Natalya Usheva, Silviya Nikolova, Yoto Yotov, Violeta Iotova

**Affiliations:** ^1^Department of Hygiene and Epidemiology, Faculty of Public Health, Medical University of Varna, Varna, Bulgaria; ^2^Department of Hygiene and Epidemiology, Medical University of Varna, Varna, Bulgaria; ^3^Institute of Endocrinology and Diabetes, Schneider Children's Medical Center, Petah Tikva, Israel; ^4^Department of Paediatrics, Medical University of Varna, Varna, Bulgaria; ^5^Department of Internal Diseases I, Medical University of Varna, Varna, Bulgaria; ^6^Department of Social Medicine and Health Care Organisation, Medical University of Varna, Varna, Bulgaria

**Keywords:** type 1 diabetes, long duration, nutrition, healthy, dietary habits

## Abstract

**Introduction:**

Nutrition education attempts to maintain and enhance good eating habits to achieve optimal metabolic control in people with type 1 diabetes (T1D). Recommendations for patients with T1D are comparable to those of the general population.

**This Study Aimed:**

To investigate dietary habits and adherence to nutritional recommendations of patients with T1D as compared with age, gender, and BMI matched people in Bulgaria.

**Methods:**

A case-control study included 124 patients with T1D with long disease duration (mean duration 25.3 ± 8.2 years) followed up at a diabetes clinic in Varna, Bulgaria for 2 years (2017–2019) and 59 controls matched for gender, age and BMI. A 24-h dietary recall method was used to assess the nutrition of both groups. A standardized questionnaire was applied to assess the frequency of food consumption (Feel4Diabtes). Height and weight were standardly measured, and BMI was calculated. Findings were compared with Bulgarian recommendations and reference values for energy and nutrient intake for healthy adults. The data were analyzed with the statistical package SPSSv21.0 and Jamovi v.22.5.

**Results:**

The nutritional characteristics of T1D men and women differ. Men with T1D had a higher intake of total carbohydrates (CHO) (*p* = 0.009), a lower intake of total fats (*p* = 0.007), and monounsaturated fatty acids (*p* = 0.029) as a percentage of total daily energy compared with the controls. Women with T1D had a different distribution of energy intake per meal compared to controls: they consumed more energy (*p* = 0.001) and a corresponding share of CHO for lunch, less for dinner (*p* = 0.015) and had a higher overall healthy diet score when compared to controls (*p* = 0.02). Adherence to dietary recommendations (e.g., CHO, total fats, saturated fat, fibers) was low in both genders, but lower in the general population compared to people with T1D.

**Conclusion:**

Our data demonstrate that people with T1D consume a healthier diet than the general population, which could be attributed to healthier diet awareness, still far from the recommendations. Introduction of annual consultations with a dietitian may improve long-term outcomes.

## Introduction

Nutrition is fundamental in the management of type 1 diabetes (T1D). Dietary counseling and guidance are integral parts of the treatment and self-management of diabetes aiming to maintain or improve nutritional and physiological health and to achieve optimal metabolic control (1, 2). Nutrition helps to prevent acute and long-term complications of diabetes and associated comorbid conditions (3, 4).

According to the International Society for Pediatric and Adolescent Diabetes (ISPAD) clinical consensus guidelines, dietary recommendations are based on healthy eating principles suitable for all children and families (5). Avoiding low-carbohydrate diet and taking 45–55 percent of total energy in the form of carbohydrates, 15–20 percent of total energy in the form of protein (more precisely 0.8–0.9 g/kg in those above 10 years), and 30–35 percent of total energy in the form of fat are some of these recommendations. ISPAD and the American Diabetes Association (ADA) are both referring to the 2015–2020 Dietary Guidelines for Americans (6) that advocate limiting of saturated fat to <10% of the total energy intake in patients with diabetes. Furthermore, the American Heart Association (AHA) (7) and the American Association of Clinical Endocrinologists (AACE) (8) both recommend limiting saturated fat to <7% of the total calories, trans-fats <1% and cholesterol <200 mg/day. Protein intake should be reduced to 0.8 g/kg in people with renal disease to avoid nephropathy deterioration (9, 10). Because establishing good glycaemic control (HbA1c 7%) is a key ADA (11) objective for patients with type 1 diabetes, balancing adequate carbohydrate intake with avoiding postprandial hyperglycemia is critical. It can potentially be handled by carbohydrate counting and insulin adjustment, as well as avoiding high fat and/or high protein meals, which may contribute to delayed hyperglycemia and the need for additional insulin dose adjustments (11).

There are no specific recommendations for patients with T1D who have a longstanding disease, and may have a double burden of disease due to the basic chronic condition and to the non-communicable disease risk that rises with age (12). Nutrition advise should be individualized, regularly evaluated, and reinforced in an intensive manner (13), and should incorporate self-management education (14). A nutritionist or dietitian should be part of the diabetes multidisciplinary team and involved in the delivery of care wherever possible (4). Many factors influence nutritional choices, which might alter over time. As a result, it is critical to gather data on the nutritional intake at regular intervals and to adapt education policies to the current demands and specific guidance for type 1 diabetes patients.

In Bulgaria, patients with diabetes are not offered a regular consultation with a dietitian. Dietary counseling is done by endocrinologists and trained endocrinology nurses. It is focused mainly on improving glycaemic control through carbohydrate matching with insulin and, to a limited degree, on healthy eating habits. It is unknown how this approach to diabetes care reflects dietary compliance with the national nutritional recommendations in Bulgaria (15).

Studies have investigated dietary intake and adherence to the recommendations in patients with T1D (16), generally reporting poor adherence for most macronutrients. For patients with longstanding T1D in Bulgaria, comparative studies with the general population have not previously been reported to the best of our knowledge. The current study aimed to investigate dietary habits and adherence to nutritional recommendations of patients with T1D as compared with age, gender, and BMI matched people in Bulgaria.

## Materials and Methods

### Design and Study Population

A case-control study was conducted over a period of 2 years (2017–2019). A sample of 124 adults (>18 years of age) with T1D with a long disease duration who were followed in the Diabetes clinic in St. Marina University hospital-Varna, Bulgaria, and 59 control subjects matched for gender, age, and body mass index (BMI) were recruited.

*Inclusion criteria* for patients and controls were: Patients with T1D for more than 15 years; healthy volunteers of the same gender, age, and BMI. The existence of any of the following conditions constitutes an *exclusion criteria*: participation in a clinical trial; significant mental impairment or other type of impediment to making an informed decision about participation; significant disability and/or immobilization; more than 3% change in body weight in the last 3 months; acute illness or condition during the study; pregnancy in women of childbearing age (in case of a delay in the regular menstrual cycle-exclusion via a pregnancy test); severe hypoglycemia or diabetes ketoacidosis in the preceding 3 months in patients with diabetes; severe confirmed microvascular diabetes complications.

Participants were interviewed using a structured questionnaire that included questions about their demographic characteristics (gender, age, and ethnicity) and socio-economic status (highest achieved educational degree, marital status, occupation, and current income). Diabetes-specific data regarding the age at diabetes onset, diabetes duration, treatment plan, control, and insulin therapy regimen was analyzed. Information on each participant's negative lifestyle behaviors, such as tobacco smoking and alcohol consumption, was collected.

A thorough clinical examination was performed together with anthropometric measurements of weight (kg), height (cm), waist circumference (cm), and the calculation of a body mass index (BMI) (kg/m^2^) and waist-to-height ratio for each participant.

Accelerometers used for 3 days were utilized to objectively evaluate physical activity level (PAL). A GCDC USB accelerometer (supported by Copyright 2011 Hookie Technologies Ltd) was used for objective measure of step counts, total amount, frequency, intensity and duration of physical activity, sedentary behavior in free living conditions for a period of 4 days.

Accelerometer was positioned on the waist and was warn full day except in case of bath or swimming. The data was uploaded and processed by online software Hookie Research Database version 1.10. The data was categorized into three physical activity level groups: based on Food and Agriculture Organization (FAO) classification of lifestyles in relation to the intensity of habitual physical activity (17). Energy intake adequateness was recalculated accordingly.

Measurement of glycated hemoglobin (HbA1c) in percent was performed after at least 12 h of fasting prior to venous blood collection. The levels of HbA1c were tested according to standardized methods in the Central Clinical Laboratory of the University Hospital “St. Marina”, Varna.

### Dietary Assessment

Data on food intake included the completion of a diet food frequency semi quantitative questionnaire—Feel4Diabetes (FFQ), and a 24-h dietary recall (24 HR).

The FFQ that we use was developed and validated with the main goal of capturing habitual dietary intake (18). The tool was applied to simplify and rate food selections, which aided in the statistical analysis. Twelve Feel4Diabetes intervention goals relating to food choices or behavior were chosen as the primary components of a Healthy diet score. Breakfast, vegetables, fruit, and berries, sugary beverages, whole-grain cereals, nuts and seeds, low-fat dairy goods, oils and fats, red meat, sweet snacks, salty snacks, and family meals were all included in these subcategories. The components were rated using the fourteen diet-related items presented in the Feel4Diabetes survey. Each component consisted of one or two questions pertaining to the frequency of intake of certain food categories or activities. These questions were used to calculate the Healthy diet score; the maximum score for each component was set based on its perceived relative importance, with a higher score indicating a better quality of diet. The overall score, which was calculated as the sum of the component scores, varied from 0 to 100, with a higher score indicating a diet of greater quality and a maximum score indicating full compliance with the Feel4Diabetes dietary objectives. This specific instrument was selected for a variety of reasons: It is composed of scored items based on food rather than nutrient intake, demonstrating dietary patterns; it is sensitive to all healthy and beneficial trends in diet; it is composed of all the relevant information available in a validated in Bulgaria questionnaire; and the Feel4Diabetes Healthy Diet Score category is significantly correlated with clinical risk factors—the blood lipoprotein profile.

The 24HR was given by a trained enumerator who gathered thorough, quantitative data on individual participants' diets by enquiring about the kind and amount of every meal and beverage consumed in the previous 24 h. The responder provided additional culinary information about each meal or beverage, such as the cooking technique and other features, as well as an estimate of the portion size ingested. The food data was matched with nutrient information from a food composition database to determine the nutrient content. The average intakes of macronutrients and micronutrients were determined. The results were compared with Bulgarian recommendations and reference values for energy and nutrient intake for healthy adults (15). According to the PAL and the corresponding reference for energy intake, participants were classified into low, adequate and high Energy intake category.

### Statistical Analysis

Descriptive statistics were reported as percentages for categorical data, mean ± standard deviation (SD) for normally distributed continuous data, and median (IQR) for non-normally distributed data. The paired comparisons between the case and control groups were done with Independent samples *t*-test for normally distributed variables and Mann-Whitney *U*-test for non-normally distributed variables. Additionally, Chi-square test was used to test for significant associations between patients and controls regarding different categorical and ordinal variables. Statistical data processing was performed using the IBM SPSS v.23 statistical package and Jamovi v.22.5. *P*-value ≤ 0.05 was considered statistically significant.

## Results

### Sample Description

A total of 183 participants – 124 with T1D, mean duration of 25.3 years without known cardiovascular diseases (67.8%) and 59 healthy controls (32.2%) were recruited for the study (Table 1). The mean age of all participants was 43.5 ± 10.1 (range 19–67 years). Both genders were equally distributed—a total of 54.1% men with no significant difference between the groups. Controls were predominantly people with higher education compared to T1D (71 vs. 50% respectively; *p* = 0.16). The majority of T1D (72.6%) and controls (81.4%) were married or living in partnership, and most self-identified as Bulgarians.

**Table 1:** Description of participants.

**Characteristics**	**Patients (n = 124)**	**Controls (n = 59)**	**χ^2^/t; p**
Male % (n)	53.2 (66)	55.9 (33)	χ^2^=0.1; p=0.73
Age y.	42.7 (10.4)	45.1 (9.2)	t=-1.6; p=0.11
Highest achieved educational degree % (n)			χ^2^ =7.9; p=0.16
Elementary/Primary	6.4 (8)	3.4 (2)	
Secondary	43.5 (54)	25.4 (15)	
College/Bachelor/Master	49.9 (62)	71.2 (42)	
Ethnicity % (n)			χ^2^ =2.5; p=0.65
Bulgarian	95.2 (118)	96.6 (57)	
Turkish	1.6 (2)	3.4 (2)	
Other	3.2 (4)	0	
Marital status % (n)			χ^2^ =5.7; p=0.13
Married or living with a partner	72.6 (90)	81.4 (48)	
Single/divorced/widowed	12.1 (15)	11.9 (7)	
Weight kg (SD)	70 (15.25)	78 (17.56)	**t=-2; p=0.04**
Height cm (SD)	169.5 (10.45)	171 (7.55)	t=-1.5; p=0.14
BMI kg/m (SD)	24 (4.04)	26 (5.05)	t=-1.5; p=0.13
Waist circumference cm (SD)	87 (13.07)	90 (15.47)	t=-1.3; p=0.74;
Physical activity level categories % (n)			**χ**^**2**^ **=7.4; p=0.024**
Low	36.4 (40)	16.1 (9)	
Middle	24.5 (27)	30.4 (17)	
High	39.1 (43)	53.6 (30)	
Smoking status % (n)			χ^2^ =1.6. p=0.45
Never smoked	37.9 (47)	28.8 (17)	
Ex- smoker	18.5 (23)	23.7 (14)	
Current smoker	43.5 (54)	47.5 (28)	
HbA1c % (SD)	8.4 (1.8)	5.4 (0.4)	**t=19.9; p<0.0001**
Duration of diabetes y. (SD)	25.3 (8.2)		

*For all qualitative parameters Chi square is used and for all quantitative data- T test was applied.*

*Data are presented as percent (n), mean (SD).*

*BMI, body mass index; HbA1c, glycated hemoglobin.*

*Statistical significance in bold*.

Table 1 provides an overview of all additional demographic, anthropometric and lifestyle characteristics of both study populations. The T1DM and control group differed significantly only regarding PAL, weight (but not BMI) and, understandably, HbA1c.

**Figure 1:**
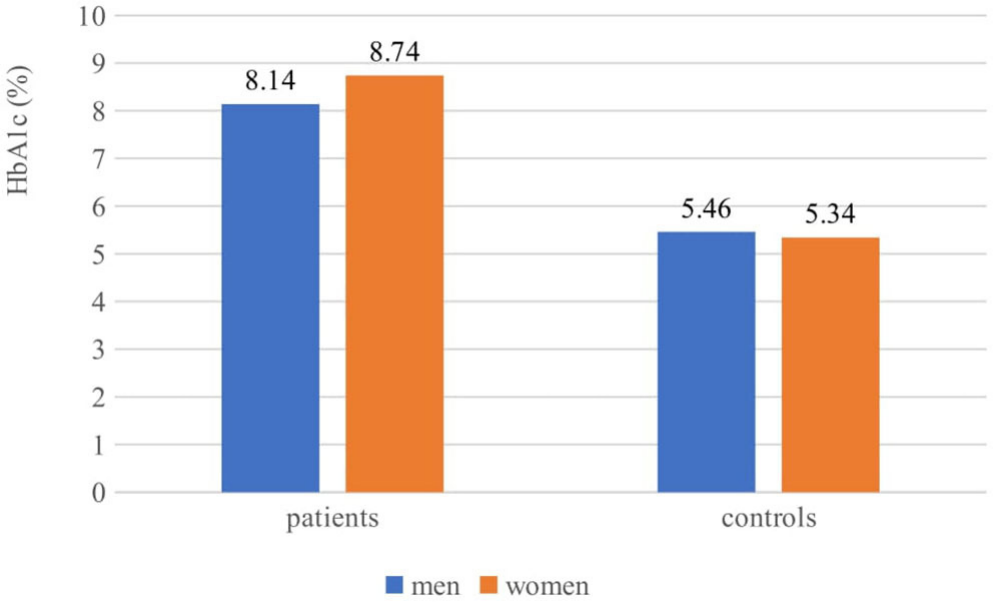
Gender based differences in glycaemic control in patients and controls.

Mean HbA1c in T1D patients was 8.4 ± 1.8% (68.5 ± 8.8 mmol/mol), 95% CI 8.11–8.73% (65.1–71.9 mmol/mol). With excellent control of diabetes (HbA1c <6%) were only 6 (4.8%), with good control (HbA1c <7%) were 14 (11.3%) of the T1D patients. There were no gender differencies in both patients and controls (Figure 1; Supplementary Figure 1). Of all patients with diabetes, 123/124 used bolus insulin and almost all −121/124 used also a basal type of insulin. The mean total insulin dose was 51.06 ± 17.33 IU, 95% CI 47.96–54.15 IU.

### Comparison of Energy and Nutrient Intake Between T1D Patients and Matched Healthy Controls

The median daily energy intake was lower in female and identical for male T1D patients as compared to controls, but with no statistical significance was found (Table 2). The proportion of patients with T1D achieving adequate Energy intake category was 25.2 and 23.7%, respectively (data not shown), but a high proportion of both patients and controls with a high BMI (>25 kg/m^2^) reported low Energy category – 54.8 vs. 66.7%, respectively (*p* = 0.70).

**Table 2:** Intake of energy and nutrients per day in T1D patients with long disease duration compared to healthy age and gender matched controls.

**Energy and nutrients** **(Units)**	**Bulgarian targets**	**Women**					**Men**					**Women Patients/** **Men Patients**
		**Patients**		**Controls**			**Patients**		**Controls**			
		**Median**	**IQR**	**Median**	**IQR**	p-value*	**Median**	**IQR**	**Median**	**IQR**	p-value*	p-value*
Total Energy intake (kcal)	>18000kcal (w) >2200kkcal (m)	1606.0	609.0	1801.0	1194.0	NS	1998.0	808.0	1866.0	1170.0	NS	< .001
Energy density (kcal/g)	NA	1.0	0.0	1.0	0.0	NS	1.0	0.0	1.0	0.0	NS	0,04
Total CHO (g)	>130	119.0	56.0	142.0	102.0	NS	157.0	99.0	101.0	88.0	0.018	0,01
Total CHO E%	45-60	33.5	12.1	30.01	14.6	NS	33.7	15.1	24.4	17.4	0.009	NS
Dietary Fibre (g)	>25	17.0	9.0	16.5	13.3	NS	18.0	12.0	16.0	10.0	NS	NS
Dietary Fibre (g/1000kcal)	>12.6	10.9	5.9	9.3	4.6	NS	9.1	5.4	8.2	3.8	NS	0,02
Total Fats (g)	NA	79.0	44.0	99.5	83.5	NS	94.0	35.0	111.0	61.0	NS	0,03
Total Fats (E%)	20-35	46.0	13.0	49.5	12.8	NS	43.0	12.0	51.0	17.0	0.007	NS
SFA (g)	NA	26.0	20.0	33.0	19.5	NS	33.0	20.8	35.0	24.0	NS	0,001
SFA (E%)	<10	15.7	6.0	14.7	6.6	NS	15.9	7.1	18.7	7.4	NS	NS
MUFA (g)	NA	23.2	17.7	29.6	26.9	NS	29.1	15.8	34.5	26.0	NS	0,05
MUFA (E%)	NA	14.45	5.6	15.1	8.8	NS	13.12	5.7	16.3	10.6	0.029	NS
PUFA (g)	NA	24.6	17.8	26.6	9.5	NS	28.7	18.7	32.2	19.1	NS	NS
PUFA (E%)	5-10	15.1	6.8	14.3	10.3	NS	11.7	7.0	14.4	10.4	NS	0,05
UFA/SFA	NA	1.0	0.4	1.1	0.6	NS	0.9	0.4	0.8	0.6	NS	0,03
PUFA/SFA	NA	1.0	0.8	1.0	0.7	NS	0.9	0.7	0.8	0.5	NS	NS
Cholesterol (g)	NA	231.0	149.0	266.0	222.0	NS	289.0	228.0	309.0	378.0	NS	0,02
Total Protein (g)	46-50 (w)/ 58-60 (m)	69.0	42.0	73.5	50.3	NS	87.5	40.8	76.0	62.0	NS	0,01
Total Protein (E%)	10-20	17.8	6.8	15.9	5.2	0.042	17.1	7.3	16.1	9.4	NS	NS
Total Animal Protein (g)	23-25 (w)/ 29-30 (m)	49.0	37.0	52.5	38.3	NS	59.5	34.5	56.0	47.0	NS	0,01
Breakfast (E%)	20	21.0	17.0	21.0	25.5	NS	20.5	27.8	23.0	25.0	NS	NS
Lunch (E%)	40	29.5	11.3	20.5	12.0	<0.001	33.0	12.0	25.0	31.0	NS	NS
Dinner (E%)	30	36.0	14.0	43.5	16.0	0.015	38.0	23.0	37.0	22.8	NS	NS
Breakfast CHO (E%)	NA	27.0	25.5	22.0	37.5	NS	20.0	28.8	24.0	24.0	NS	NS
Lunch CHO (E%)	NA	26.0	12.0	14.5	19.8	0.015	29.0	20.5	25.0	32.0	NS	NS
Dinner CHO (E%)	NA	29.0	19.0	26.5	18.8	NS	32.0	27.0	33.0	30.0	NS	NS
Alcohol consumption (g)	20 (m) /10 (w)	9.0	5.0	9.5	5.8	NS	12.0	7.0	10.0	6.0	NS	0,00
Feel4diabetes Score	NA	62.0	16.3	53.0	10.5	0.002	56.5	15.0	56.0	13.0	NS	0,01

* *Mann–Whitney U test.*

*CHO-carbohydrates; E%- energy percent g-grams kcal- kilocalories m-men; MUFA- monounsaturated fatty acids; NS, not significant; PUFA, polyunsaturated fatty acids; SFA, saturated fatty acids; UFA, unsaturated fatty acids; w, women*.

The median intake of carbohydrates was lower than the range of 45–60 E%, and closest to the adequate in male patients (33.7 E%; 157.5 g>130 g). Although higher for T1D, the median for dietary fiber did not reach the recommended intake of 25 g/day, and was lowest again in male controls (16 g/day). Adjusted for total energy, fibers intake was highest in female T1D patients compared to controls (~11 g/1,000 kcal). All groups had adequate to the recommended intake of proteins (~17 E% in patients with diabetes vs. ~16 E% in the control group), while the absolute amount of protein (in g) was higher than the recommendation, and the type of protein was mainly animal protein (>50% of total protein). All groups ingested higher than the reference level of total fat (>20–35 E%) and saturated fatty acids (SFA) >10 E%, the highest values reported by male controls—total fat 51 E% and SFA 18.7 E% (*p* = 0.007). Compared with the Bulgarian recommendation for polyunsaturated fatty acids (PUFA) intake of 5–10 E%, the adherence of all groups was acceptable, but lowest for male controls.

Both genders of T1D patients had higher adherence to Energy intake distribution per meal with closer to the recommended amount for Lunch (recommended ~40 E%) and Dinner (recommended ~30 E%) with the least fluctuations of carbohydrates (CHO) E% consumption in women patients. The median alcohol intake for the participants was within the recommendations in male T1D participants demonstrating a 17% lower intake as compared to controls (10 vs. 12 g).

In a subgroup comparison of female and male patients, it was determined that women had significantly superior eating habits, particularly with the consumption of saturated (26 vs. 33 g, *p* = 0.001), unsaturated fats, fiber, and animal protein, as well as alcohol consumption (Table 2).

**Figure 2:**
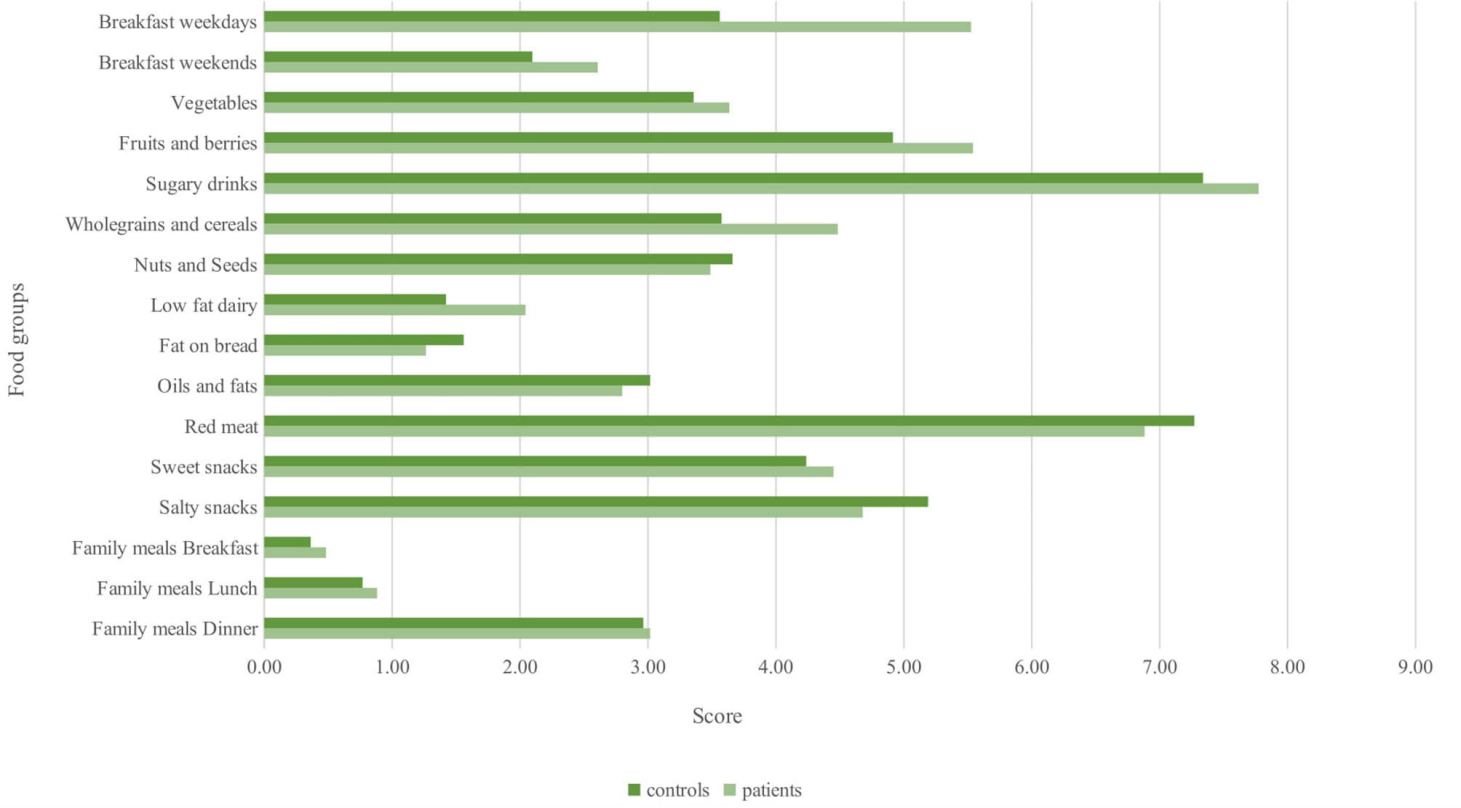
Comparison between T1D patients and controls of items included in Feel4Diabetes healthy score.

Applying the Feel4diabetes scoring system, most of the food groups scored higher in patients (Figure 2), except for snacks and fats. Statistical significance was reached only for the regular presence of breakfasts—on week days (median 7 vs. 4 points, *p* = 0.001) and weekends (median 3 vs. 2 points, *p* = 0.002), T1D subjects vs. controls. The clear trend for female patients to have a higher FFQ than male patients was once again evident (62.0 vs. 56.5, *p* = 0.01).

## Discussion

The current study revealed that both T1D patients and controls are not following the Bulgarian dietary guidelines (15) for energy and macronutrient consumption. Nevertheless, patients had some beneficial trends when compared to controls. Total fats, particularly SFA and cholesterol, were high in T1D patients but even higher in controls, with only total fats in men- T1D vs. controls, reaching significance. These findings demonstrate an even worse trend than that observed in the 2014 Bulgarian representative survey (19). Similar findings were observed in previous studies on the diets of patients with T1D since adolescence, where total fat and saturated fat intake surpassed recommendations, even when energy intake was low (2, 20–22). Historically, dietary approaches have focused on lowering saturated fatty acids (SFA) and dietary cholesterol. Diets that have <7% SFA and <200 g of dietary cholesterol a day have been shown to improve lipids and other cardiovascular disease (CVD) risk factors when compared to diets with more SFA and less cholesterol. In recent years, however several studies have revealed that for CVD risk reduction, the quality of fat (the kind of fatty acids) is more important than the quantity of fat.

Interestingly, CHO consumption was low in all participants, both as a percentage of daily energy and as an absolute intake in grams, and reached a significant difference in men with T1D compared to controls. Previous research also discovered a lack of compliance with the dietary advice for CHO (2, 16, 20) with a tendency to consume less CHO. While dietary recommendations are evidence-based and the existing data does not support a low-CHO diet, female patients and male controls tended to have a low-CHO diet, less than the recommended minimum of 130 g/day. Contrary to the popular belief, a low-carbohydrate diet can interact with the hyperglycaemic consequences of fat and protein consumption (23). There is evidence that such diets may potentially be harmful to the patient's health. They can cause acute hypo- or hyperglycemia, worsen the predictability of the diet on glycemia, increase the likelihood of ketosis, and deplete systemic glycogen reserves. Although the long-term effects of a low-carbohydrate diet are unknown, potential concerns include changes in lipid profiles, nutritional deficiency, CVD problems, and nephrolithiasis (23).

On the other hand, fiber intake is also lower than recommended in our study (4). Neither patients nor controls met the Bulgarian requirement of 25 g fiber per day as well as fiber density (12.6 g of fiber per 1,000 kcal), which is consistent with previous observational research (22, 24). Perhaps the recommended dietary fiber consumption is impossible for the majority of people, given the country's traditional diet, which continues to vary seasonally and the availability of fiber-rich foods during the winter, as well as the diet's westernization during the last 30 years. There is widespread agreement among nutritional societies that increasing fiber intake, particularly from whole grain cereals, is associated with a reduction in CVD-specific and all-cause mortality in T1D patients and also improves glycemic control, with a significant reduction in HbA1c following fiber intake increases (4). Long-term compliance with such high fiber intakes, on the other hand, may be difficult in daily practice (21).

The participants' median protein intake stayed within the recommended range as a percentage of energy, but was significantly higher as an absolute intake in grams. Proteins have been shown to defend against the onset of hypoglycemia (5). Although the participants stayed within the recommended protein consumption range (10–20% E) for people with diabetes who do not have abnormal albuminuria, there was a definite tendency toward increased animal protein and decreased plant protein, respectively. This could have been driven in part by patients' focus on insulin-CHO ratio and desire to reduce CHO, which resulted in an increase in animal protein intake together with total and saturated fat in their diets, as these dietary components are abundant and co-present in animal foods. The current findings corroborate those of several earlier observational studies (25, 26). The within-recommended intake of protein is important, as increased protein consumption impairs the synthesis of anti-insulin hormones such as glucagon, resulting in a postprandial rise in blood glucose levels, as well as cortisol secretion, resulting in insulin resistance and elevated postprandial blood glucose levels (27).

Our findings indicated that dinner was the principal meal of the day in the Bulgarian population- the E percent from dinner meals was higher than recommended in both patients and controls, implying that supper is the primary meal of the day. This may pose a difficulty for insulin dose calculation since dinner has the worst postprandial control of all meals (28), with a glucose response lasting up to 6 h after the meal. This is of particular concern because, together with its characteristics as a protein- and fat-dense meal, it may result in an even higher increase in postprandial glucose concentrations and for long hours during the night. Thus, we can hypothesize that late postprandial glucose increases are more pronounced.

With regards to healthy eating habits according to the Feel4Diabetes score (18) we observed a significant difference in overall score between men and women with T1D, indicating a greater awareness in female patients of the importance of the frequency of consumption of various food groups. However, upon closer examination of the individual items, the difference was primarily for breakfast frequency. Breakfast inclusion and the suggestion of at least three meals per day are two of the recommendations for healthy eating in Bulgaria.

Although the Feel4Diabetes score was designed largely for the purpose to prevent type 2 diabetes, it represents both favorable (higher score) and unfavorable nutritional (lower score) trends, which are critical in the management of T1D as well. The Healthy Diet Score was found to be strongly, if moderately, linked with clinical risk variables such as HDL- and LDL-cholesterol and triglycerides, all of which are critical for long-term T1D treatment. In our study, we observed a significant difference in overall score between men and women with T1D, indicating a greater awareness in female patients of the importance of the frequency of consumption of various food groups. However, upon closer examination of the individual items, the difference was primarily for breakfast frequency. Breakfast inclusion and the suggestion of at least three meals per day are two of the recommendations for healthy eating in Bulgaria. Comparative studies on meal and snack frequency are nearly non-existent. Nonetheless, a recent study discovered that when single meal occasions were compared to corresponding HbA1c levels, both breakfast and lunch were associated with improved glycemic management (29).

Among the study's strengths is the large sample size for Bulgaria, which allowed for the collection of comprehensive data on energy, nutrient intake assessment, meal/snacking frequency, and associated carbohydrate intake. The meal pattern and energy and nutrient consumption of patients with long-term T1D are of particular interest, as the majority of nutrition research focuses on T2D and, when T1D patients are included, does not differentiate between recently diagnosed and long-term disease duration patients. For the first time, the nutrition of Bulgarian patients was investigated. Another strength was the use of a validated in the country tool to collect dietary data, as well as the assessment of potential discrepancies in eating patterns between patients and control groups, which reflected nutrition in the general community. Additional value was contributed by using two different approaches to assess nutrition: a 24-h recall and a FFQ.

Though we were unable to reassess nutrition using a second 24-h recall, we can presume that all type 1 diabetes patients for whom solely dietary data was available were assessed using an FFQ. The degree of plausibility attained in energy reporting was poor and in a high proportion of patients it did not correspond to BMI, indicating that participants may be underreporting. Nonetheless, the obvious trends outweigh the underreporting. We were unable to recruit a larger number of controls in our study, and the ratio of controls to patients was 1:2, which may have contributed to the uncertainty regarding the nutrition of controls but not so much to the eating habits of T1D patients. Lastly, the cross-sectional nature of the study design limits the generalizability of the study.

## Conclusion

In conclusion, our study found that both patients with T1D and controls frequently do not adhere to the country's dietary recommendations. Bulgarian patients with T1D consume significantly less CHO and dietary fiber and have a higher total fat intake, which contributes to possible poor glycemic control and may increase the risk of late metabolic problems. Still, people with T1D consume a healthier diet than the general population, which could be attributed to healthier diet awareness, still far from the recommendations. These findings lend support to the notion that dietitian-assisted dietary counseling may result in an increase in patient adherence to dietary guidelines, as well as improve diabetes control and reduce CVD risk factors and other diabetic complications. Dietary education for people with T1D should place a higher emphasis on efforts to improve the overall quality of the diet and, consequently, on adherence to current dietary recommendations for diabetes management and metabolic control.

## Data Availability Statement

The original contributions presented in the study are included in the article/Supplementary Material, further inquiries can be directed to the corresponding author/s.

## Ethics Statement

The studies involving human participants were reviewed and approved by Ethical Committee at the Medical University of Varna. The patients/participants provided their written informed consent to participate in this study.

## Author Contributions

YY and VI conceptualized and supervised the study. TC and KT have been involved in the design of the study. RP conceived the idea. LD selected and retrieved relevant papers. RP, LD, and MG-K drafted this review. NU and SN evaluated the statistical analysis. All authors revised and approved the final manuscript.

## Funding

This study was supported by a research grant N 17022/2017, from the Fund “Science”, Medical University of Varna.

## Conflict of Interest

The authors declare that the research was conducted in the absence of any commercial or financial relationships that could be construed as a potential conflict of interest.

## Publisher's Note

All claims expressed in this article are solely those of the authors and do not necessarily represent those of their affiliated organizations, or those of the publisher, the editors and the reviewers. Any product that may be evaluated in this article, or claim that may be made by its manufacturer, is not guaranteed or endorsed by the publisher.

## Abbreviations

24HR: 24-h dietary recall
AACE: American association of clinical endocrinologists
ADA: American diabetes association
AHA: American heart association
BMI: Body mass index
CHO: carbohydrates
CI: confidence interval
CVD: cardiovascular disease
E%: energy percent
FAO: food and agriculture organization
Feel4Diabtes: feel for diabetes diet score
FFQ: food frequency questionnaire
g: grams
HbA1c: glycated hemoglobin
IQR: interquartile range
ISPAD: international society for pediatric and adolescent diabetes
IU: international units
kcal: kilocalories
MUFA: monounsaturated fatty acids
NS: not significant
PAL: physical activity level
PUFA: polyunsaturated fatty acids
SD: standard deviation
SFA: saturated fatty acids
T1D: type 1 diabetes
UFA: unsaturated fatty acids.
