# Supplementary figures and images for: Dietary Behavior and Compliance to Bulgarian National Nutrition Guidelines in Patients With Type 1 Diabetes With Longstanding Disease
Source: Front Nutr. 2022 Jul 8;9:900422. doi: 10.3389/fnut.2022.900422 (PMC9305306; doi:10.3389/fnut.2022.900422)

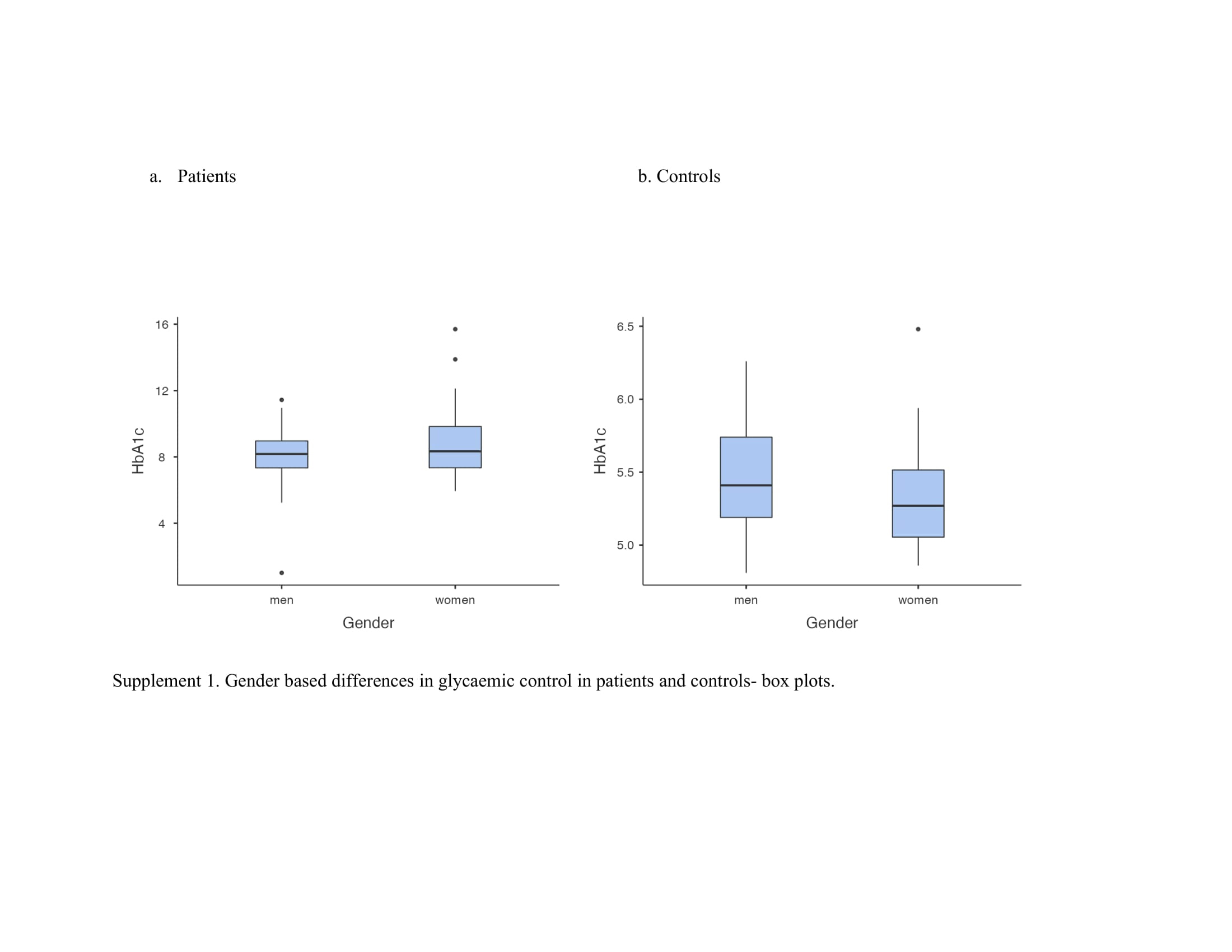

Supplement: Supplementary file 1 [file Image_1.jpg]
